# A highly sensitive underwater video system for use in turbid aquaculture ponds

**DOI:** 10.1038/srep31810

**Published:** 2016-08-24

**Authors:** Chin-Chang Hung, Shih-Chieh Tsao, Kuo-Hao Huang, Jia-Pu Jang, Hsu-Kuang Chang, Fred C. Dobbs

**Affiliations:** 1 Department of Oceanography, and Asia-Pacific Ocean Research Center, National Sun Yat-Sen University, Kaohsiung, 80424, Taiwan; 2 Department of Ocean, Earth and Atmospheric Sciences, Old Dominion University, Norfolk, VA, 23529 USA; 3 Taiwan Ocean Research Institute, Kaohsiung, Taiwan

## Abstract

The turbid, low-light waters characteristic of aquaculture ponds have made it difficult or impossible for previous video cameras to provide clear imagery of the ponds’ benthic habitat. We developed a highly sensitive, underwater video system (UVS) for this particular application and tested it in shrimp ponds having turbidities typical of those in southern Taiwan. The system’s high-quality video stream and images, together with its camera capacity (up to nine cameras), permit *in situ* observations of shrimp feeding behavior, shrimp size and internal anatomy, and organic matter residues on pond sediments. The UVS can operate continuously and be focused remotely, a convenience to shrimp farmers. The observations possible with the UVS provide aquaculturists with information critical to provision of feed with minimal waste; determining whether the accumulation of organic-matter residues dictates exchange of pond water; and management decisions concerning shrimp health.

Shrimp is one of the world’s most popular seafoods. Global production of farmed shrimp was approximately 3.5 million tonnes in 2013^1^. Two of the most important issues in shrimp aquaculture are welfare (or health) and pond management (feed and water quality). Shrimp welfare can be abetted in part by using specific pathogen free (SPF) shrimp when stocking farms with postlarvae. Issues of pond management, however, are more difficult to control during the normal growth period of shrimp (from postlarvae to marketable shrimp), 5 to 8 months or longer. Shrimp growth is highly influenced by the amount of food available, however, overfeeding not only wastes feed, but the excess organic-matter residue results in poor water quality, increasing aquaculture costs. Therefore, it is crucial to monitor the provision of feed and feeding extent in farmed shrimp ponds.

Water transparency in shrimp ponds is largely affected by high concentrations of suspended particulate matter attributable to bioflocs, phytoplankton, and residues of feed and shrimp waste^2^,^3^,^4^; visibility is often less than 100 cm. It is a considerable challenge, therefore, to conduct an underwater visual inspection in such highly turbid waters. Traditionally, checks of feeding trays have been used to implement feed control, but daily checking of feed trays requires skilled labor; furthermore, feed tray analysis is subjective^5^. Acoustic control and imaging sonar improve feeding productivity within fish cage and shrimp farms, and measurement of swimming patterns and body length of cultured fish^5^,^6^,^7^, but the technologies cannot deliver real-time images of shrimp feeding and welfare in the turbid benthic layer.

Underwater video systems have been used since the 1950s to monitor *inter alia* marine plankton, fish behavior, and freshwater biodiversity^8^,^9^,^10^,^11^. For the most part, however, these video systems have been operated in clear-water environments, including coastal fish cages. In this study, we developed and used a sensitive, underwater video system (UVS) to monitor benthic conditions and shrimp feeding in the turbid, low-visibility waters of shrimp ponds. The specific issues we addressed were to: (1) understand shrimp feeding, because excess feed incurs waste and causes poor water quality; (2) examine the benthic surface for fecal pellets and excess organic matter, both of which may result in increased pathogen concentrations and a fouled bottom; (3) visualize shrimp in turbid waters to estimate their size and behavior, the latter because it can indicate water quality and infection by pathogens; (4) provide *in situ* underwater images to fish farmers to reduce their economic loss during extremely cold weather. The sensitive UVS we describe here has strong potential to be used in commercial shrimp and other aquaculture operations.

## Results and Discussion

### Monitoring shrimp feeding in turbid water

**Figure 1:**
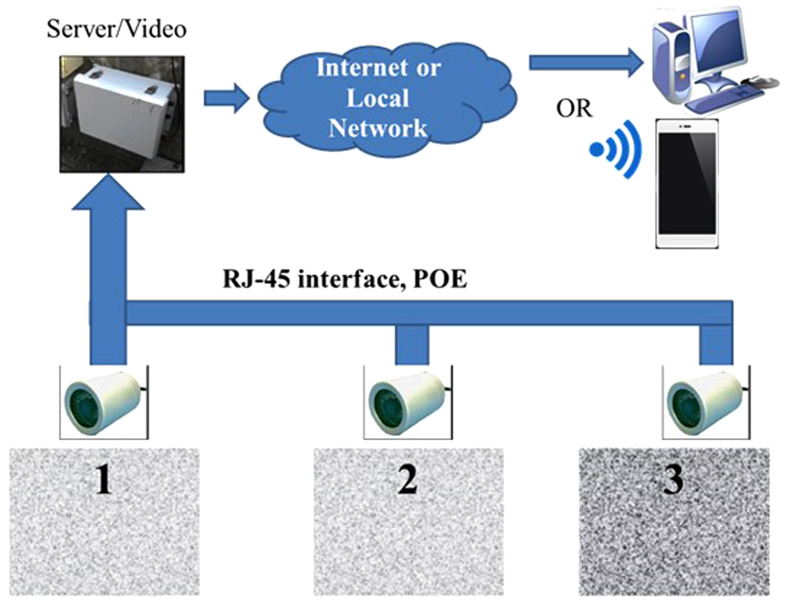
The underwater video system (UVS) used in shrimp ponds of different turbidities at the campus of National Sun Yat-Sen University. Pond 1: low turbidity water (TSM = 4.4~6.4 mg/L, mean value = 5.6 ± 0.9 mg/L, n = 4), Pond 2: low turbidity water (TSM = 5.2~8.5 mg/L, mean value = 6.8 ± 1.4 mg/L, n = 4), Pond 3: high turbidity water (TSM = 27~73 mg/L, mean value = 48 ± 17 mg/L, n = 5). PoE = Power over Ethernet. TSM: total suspended matter.

**Figure 2:**
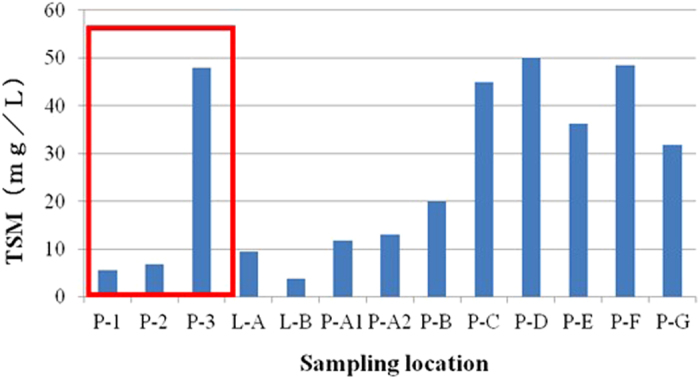
Concentrations of total suspended matter (TSM) in shrimp ponds from different locations. P-1 to P-3 (within red rectangle) represent concentrations of TSM in shrimp ponds 1, 2, and 3 respectively. Other TSM concentrations are from lakes (labeled L-A and L-B) or shrimp/fish farms (labeled P-A1 to P-G; PA-1 and PA-2 represent duplicate sampling at pond A) in southern Taiwan.

In most countries of Southeast Asia, including Taiwan and China, shrimp farms usually grow animals at high densities (>100 shrimps m^−2^) in closed greenwater ponds or biofloc systems. The water used, therefore, often is highly turbid due to suspended material, phytoplankton, or both. Usually, visibility is approximately 100 cm, but sometimes less than 50 cm (Fig. 1). Mean values of total suspended material (TSM) in the three shrimp ponds (P1, P2, P3) on the NSYSU campus ranged between 4.4 and 48 mg/L, consistent with the range in lakes and commercial shrimp ponds across southern Taiwan (3.8 to 50 mg/L, Fig. 2). Turbidity in the UVS images presented here, therefore, is representative of conditions in Taiwanese shrimp farms.

**Figure 3:**
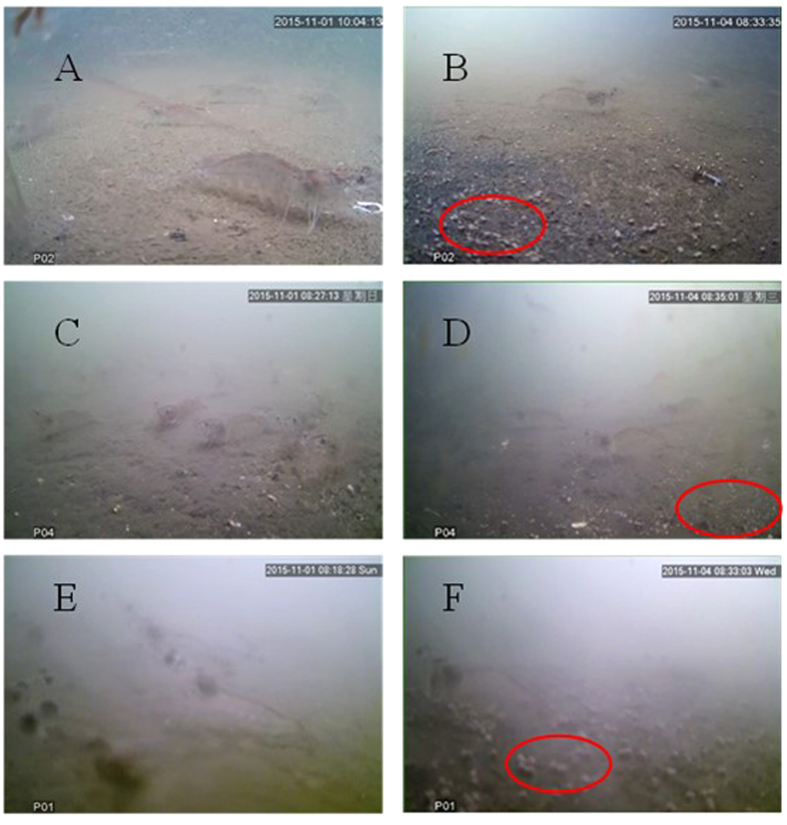
Images showing formulated feed pellets (within red circles) upon the bottom of ponds having different turbidities (panels **A,C,E**: before feeding at ponds 1, 2 and 3; panels **B,D,F**: during feeding at ponds 1, 2, and 3).

The low-light intensity characteristic of turbid water has proven problematic for camera systems in the absence of auxiliary lighting^8^. The UVS, however, functions well even at 15 lux or less. Selected UVS images (snapshot mode) serve to demonstrate the system’s ability to visualize evidence of shrimp feeding, even in aquaculture ponds. Figure 3 contains images of shrimp ponds 1, 2, and 3 before (panels A,C,E) and after (panels B,D,F) provision with formulated feed pellets, indicated by red circles. In low-turbidity ponds 1 and 2 (panels A-D), shrimp are visible before and during feeding. Even in pond 3, characterized by high TSM and bioflocs, feeding pellets are visible on the sediment surface (panels E,F).

Traditionally, shrimp farmers determine the rate and extent of food consumption using a feeding tray to which pellets are added. The tray is lowered into the pond, retrieved after some tens of minutes, and inspected to infer shrimp feeding. This method relies heavily on the farmer’s experience and can be misrepresentative in cases of feeding lag or over feeding during the short time the tray is deployed. In contrast, clear, real-time images of food pellets and shrimp feeding behavior provided by the UVS allow a temporally integrated fine-tuning of food supply. Less well resolved images taken in highly turbid waters (e.g., Pond 3) also provide important information, unavailable from feeding-tray assessments, permitting farmers to adjust the amount of feed accordingly. Overall, therefore, the UVS largely improves on the traditional feeding-tray method to monitor shrimp feeding.

Acoustic control systems, used to improve feeding productivity in fish cages and shrimp farms^5^,^6^, are suitable for clear-water systems or shrimp ponds of large size (e.g., >10 ha). Acoustic control may not be cost effective in Taiwan, Thailand, or China, however, because most shrimp farms in those countries range in size from approximately 0.16 to several ha^2^. In addition, a high percentage of intensive shrimp farms in Taiwan and Thailand are owned by small-scale operators^2^ who unlike their large-scale counterparts, do not require or cannot afford pioneering instrumentation. Although the UVS is not without cost, it provides not only real-time feeding information, but additional insights as well (next section).

### Evaluating excess organic matter, shrimp size, and shrimp health

**Figure 4:**
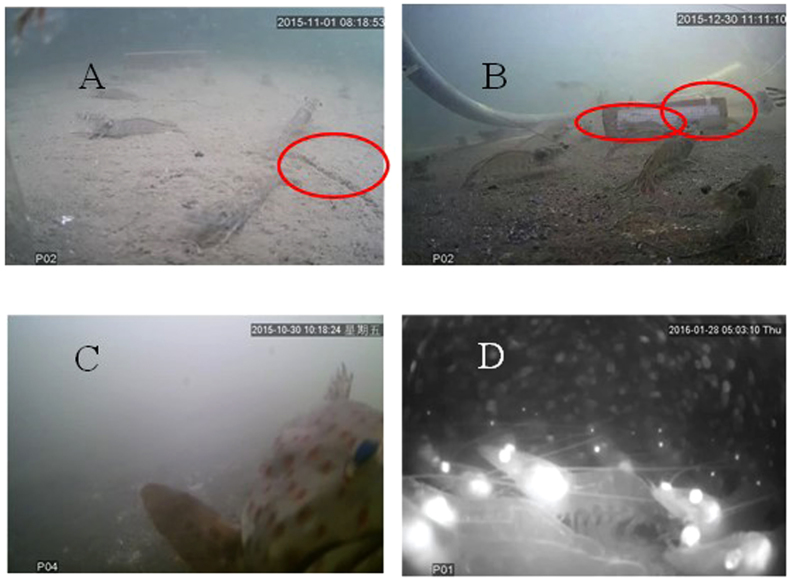
(**A**) A furrow in the surface layer of detritus (red oval) indicates the thick layer of organic matter on the sediments in shrimp pond 1. (**B**) Two shrimp immediately in front of a white ruler (scale bar = 15 cm) affixed to a brick. (**C**) Image of a grouper in a turbid pond. (**D**) Image of multiple shrimp at night. At light levels <15 lux, black and white images, not color, are returned.

Jackson *et al.*^3^ reported that sediments in shrimp ponds contain 14% (in terms of total nitrogen) of formulated food delivered to the shrimp. This excess organic matter, plus that from shrimp feces, phytodetritus, etc., provides a substantial nutrient source for bacteria, some of which may be pathogenic. To reduce bacterial load overall and keep water quality high in Thailand, shrimp ponds are heavily aerated to concentrate particulate organic waste at their center, rather than have it distributed randomly across the pond bottom^2^. Confirming a concentrated distribution, however, has previously not been easily accomplished. The UVS makes possible rapid detection of waste-rich organic sediment in turbid waters or greenwater systems, for example, the thick layer of organic detritus and formulated feed pellets seen in Fig. 4A,B. With clear imagery of the benthic surface, shrimp farmers can decide when and to what degree to clean pond sediments or exchange fouled pond water with fresh seawater.

**Figure 5:**
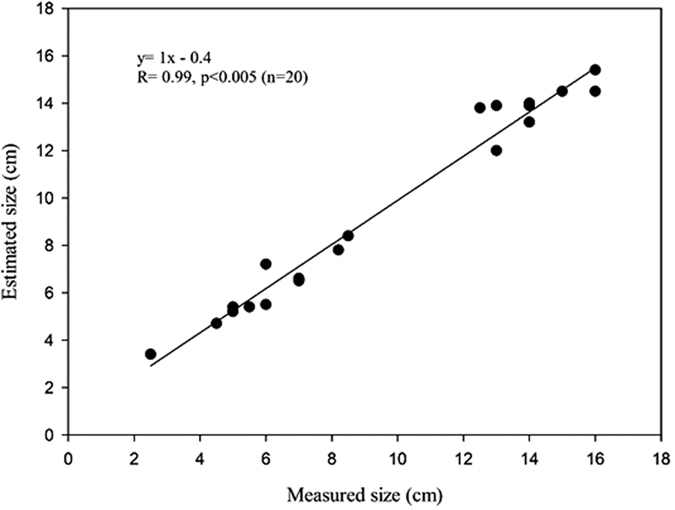
Relationship between estimated length (from video image) and measured length of shrimp.

The UVS also can be used to estimate shrimp size *in situ*, precluding the need to collect them with nets for length measurements. For example, with reference to a ruler attached to a brick, we estimated two shrimp in Fig. 4B each to be approximately 10 cm long. In a calibration exercise, these estimates correlated strongly with manual measurements of shrimp length: y = 1.04x-0.4, r = 0.99, p < 0.005, n = 20, where y is estimated size (cm) and x = measured size (cm) (Fig. 5). Deployment of a standard-sized mesh (e.g., from netting) on the sediment surface would facilitate size estimates of multiple shrimp and account for changing perspective with distance from the camera.

One can assess the health status of shrimp according to their swimming and feeding behaviors and body transparency^12^. The resolution provided by the UVS (see video of healthy shrimp in Supplemental Material) makes possible detailed assessment of behavior and inspection of internal organs. Abnormal behaviors typically caused by poor water quality or disease, including lethargy, sluggish swimming, and spiral swimming, can be easily seen. Given its ability to monitor shrimp feeding, behavior, and internal organs, even in turbid waters, the UVS can serve farmers as an early warning system to detect unhealthy shrimp, making possible an immediate response to overfeeding, disease, or poor water quality.

### Providing real-time underwater images to aquaculture farmers

Asia is the biggest aquaculture production area in the world and 70% of global aquaculture products are produced by China and Taiwan^13^,^14^. In January 2016, a lethal cold snap in those two regions killed aquaculture stock including fish, clams, and shrimp^15^. In Taiwan alone, losses were estimated at more than $100M US^16^. Fish farmers were able to promptly harvest some types of cold-stunned fish, e.g., milkfish and tilapia, and reduced their economic loss. Losses of demersal fish and shrimp were profound, however, in large part because they could not be seen in high-turbidity water. In particular, 2- to 3-year old groupers weighing 10 to 30 kg were killed but unseen until they floated to the surface of fish ponds several days after the extremely cold weather. They were spoiled and unharvestable. In cases such as these rare cold snaps (ca. once per decade), the UVS could supply fish farmers with images pertinent to making harvest decisions to save their stocks or at least to reduce their property loss (Fig. 4C).

### Advantages of the UVS and its future improvements

Struthers *et al.*^17^ recently reviewed commercial, portable, underwater cameras that have been used under relatively clear conditions. Compared to the UVS, such cameras have limitations: (1) They are battery powered (battery life time 2~3 hours), whereas the UVS uses a PoE cable; no battery re-charge is needed. (2) Most commercial cameras cannot provide real-time images; the UVS can provide them from several cameras (up to 9) simultaneously. (3) Each camera in the UVS can be operated manually or controlled by computer or smart phone via internet and images can be stored for 5 days (up to a 10-day capacity). (4) Functions of the UVS can be expanded to include sensors for temperature, salinity, pH, dissolved oxygen, etc. (5) The UVS can be operated in fish ponds long-term (24 hours/day for more than 1 year) and the depth of deployment can reach 1000 m if needed (a different housing is used). (6) The resolution of the UVS can be upgraded if budget is not a consideration.

Is there an effect on shrimp behavior at very low light levels (<15 lux) when the IR bulbs are activated? Some fish are sensitive to IR light in highly turbid habitats^18^, but shrimp likely are not, based on reports of their spectral sensitivity. Vision in the penaeid shrimp, *Sergestes similis*, has a single peak of sensitivity from 480 to 520 nm^19^ (Lindsay *et al.*^19^). Frank and Widder^20^ tested vision in 12 species of mesopelagic crustaceans, including shrimp, and found all had a strong peak within the range 470 to 500 nm. In investigating the effects of different light sources on growth of *Litopenaeus vannamei*, You *et al.*^21^ showed the shrimp grew rapidly when illuminated by a metal halide lamp (MHL). The spectrum of a typical MHL, however, is between 385 nm and 674 nm, suggesting only visible light affects shrimp growth. Finally, Sanudin *et al.*^22^ concluded that lighting conditions, including IR wavelengths, did not affect feeding by post larvae (PL10 and older) of *Penaeus vannamei*. Therefore, although we did not run IR sensitivity experiments, the literature suggests shrimp are insensitive to IR light.

Judicious siting of the video cameras, which currently “see” only a fixed area, can allow assessment of the pond overall. To increase coverage for large shrimp ponds, farmers could install additional cameras; one server can control nine cameras based on current technology. In the future, we envision rotating UVS, even mobile ones, to increase the area monitored. Such systems could be connected with software to monitor and control the ponds’ environment based on real-time imagery. That is, the UVS is a first step in developing fully automated systems to detect the amount of food available to the shrimp, provide food when needed, assess the degree of accumulation of sediment in the ponds, and even differentiate between low light levels caused by turbidity vs absence of sunlight. We understand conceptually how machine learning, integrated with the UVS, can achieve these goals and hope in the future to investigate them.

## Materials and Methods

### Underwater video system (UVS)

The underwater video system (UVS) contains a 500-GB hard drive (expandable up to 2-TB), a server, and a power supply (each camera operates at 12 V and 500 mA) supporting three custom manufactured underwater cameras; the system can be configured with as many as nine cameras (Fig. 1). Each camera is emplaced autonomously. The real-time image from each is processed by a built-in digital signal processor (DSP) and sent to a server through a power over Ethernet (PoE) cable, then transported to an internet account via a wireless network.

Each camera has two 3-watt infrared (780 nm) LED bulbs, contained within a waterproof, aluminum alloy (Aluminum 6061-T6) housing with an acrylic window. Cameras were fixed approximately 20~30 cm above the bottom of shrimp ponds to monitor shrimp activity, feeding status, welfare, and benthic sediments. The UVS captures color images when the light intensity is higher than 15 lux; below that level, the infrared LED bulbs are controlled by an IR-Cut sensor and turn on automatically, generating black and white images. The system is reliable in seawater shrimp ponds (salinity ranging from 30 to 34); the UVS operated at depths of 1.2 to 1.8 m for more than 12 months. The acrylic window needs to be cleaned of biofouling every 3 to 4 days. On sunny and most cloudy days, the UVS operates in color mode because the light intensity at the bottom of the ponds is approximately 790 to 1,580 lux (about 1% of surface light intensity, based on annual sunlight intensity data in southern Taiwan ( www.cwb.gov.tw). Therefore, the UVS can be used in low transparency waters during the daytime without extra light sources. The underwater images can be accessed remotely by a smart phone or through the internet.

### Video camera specifications

The camera (OV2710) is a true full HP (1080p) CMOS image sensor designed specifically to deliver high-end HD video to a digital video camcorder with a display resolution of 1920 × 1080 pixels, operating at 30 frames per sec (for more details, see the OV2710 commercial website, OV2710 product brief). Built with Omni Vision’s proprietary 3 μm OmniPixeI3-HS high sensitivity pixel technology, the OV2710 delivers low light sensitivity of 3300 mV/lux-sec, S/N ratio of 39 dB, dark current of 10 mV/sec, and a peak dynamic range of 69 dB, which in combination allow the camera to operate in conditions ranging from strong light to nearly complete darkness (below 15 lux), such as in highly turbid aquaculture ponds, even at night, so that 24 hour surveillance of shrimp or fish is possible (Fig. 4D).

### Measurement of total suspended matter (TSM)

Concentrations of TSM in lakes and aquaculture ponds in southern Taiwan were measured using the method of Hung *et al.*^23^. Briefly, 20 to 150 ml of water was filtered through a tared 25 mm polycarbonate filter (pore size = 0.4 μm), then the filter was gently rinsed with 15 to 20 mL of Milli-Q water to remove salts. The filter was dried, weighed on a micro balance, and the mass of suspended matter was calculated by difference.

### Application of UVS in aquaculture ponds of different turbidity

We conducted observations in three shrimp ponds on the campus of National Sun Yat-Sen University from June 2014 to November 2015. Each pond contained between 40 to 70 tonnes of seawater and supported at a minimum several thousand white shrimp, *Penaeus vannamei*. Shrimp feed pellets were evenly dispensed three times daily. TSM concentrations in ponds 1, 2, and 3 ranged from 4.6 to 6.4, 5.2 to 8.2, and 27 to 73 mg/L, respectively (Fig. 2).

We observed shrimp behavior and feeding via real-time UVS. The UVS image stream was recorded, allowing review of previous activities (see video in Supplemental Materials). We evaluated benthic surface conditions with respect to feed pellets, fecal pellets, and excess organic matter. We estimated the length of shrimp that swam or crawled adjacent to a ruler (15 cm long) affixed to a brick. We calibrated our length estimates using shrimp individually measured by hand, placed in a large tank filled with shrimp pond water, and measured again using the UVS and a ruler.

## Additional Information

**How to cite this article**: Hung, C.-C. *et al.* A highly sensitive underwater video system for use in turbid aquaculture ponds. *Sci. Rep.* **6**, 31810; doi: 10.1038/srep31810 (2016).

## Supplementary Material

Supplementary Information

Supplementary Video 1
