# Supplementary material for: A highly sensitive underwater video system for use in turbid aquaculture ponds
Source: Sci Rep. 2016 Aug 24;6:31810. doi: 10.1038/srep31810 (PMC4995459; doi:10.1038/srep31810)
Supplement: Supplementary Information [file srep31810-s1.pdf]

A highly sensitive underwater video system for use in turbid aquaculture ponds

Chin-Chang Hung[1,2*], Shih-Chieh Tsao[1], Kuo-Hao Huang[1], Jia-Pu Jang[3], Hsu-Kuang Chang[3], and Fred C. Dobbs[2]

1. Department of Oceanography, and Asia-Pacific Ocean Research Center, National Sun Yat-Sen University, Kaohsiung, 80424, Taiwan.
2. Department of Ocean, Earth and Atmospheric Sciences, Old Dominion University, Norfolk, VA, 23529 USA
3. Taiwan Ocean Research Institute, Kaohsiung, Taiwan.

The video in supplemental material:

The video shows a school of healthy shrimp swimming, eating, and searching for formulated food pellets. Furthermore, the UVS provides clear views of the shrimps' antennae, guts, and stomachs (in this case, full of food). Given its ability to monitor shrimp feeding, behavior, and internal organs, even in turbid waters, the UVS can serve farmers as an early warning system to detect unhealthy shrimp, making possible an immediate response to overfeeding, disease, or poor water quality. Bioflocs are visible flowing toward the left from top right.
